# Patterns of crop cover under future climates

**DOI:** 10.1007/s13280-016-0818-1

**Published:** 2016-09-23

**Authors:** Luciana L. Porfirio, David Newth, Ian N. Harman, John J. Finnigan, Yiyong Cai

**Affiliations:** 1 Oceans & Atmosphere, The Commonwealth Scientific and Industrial Research Organisation (CSIRO), Yarralumla, ACT 2601 Australia; 2 Centre for Applied Macroeconomic Analysis, The Australian National University, JG Crawford Building, 132 Lennox Crossing, Acton, ACT 0200 Australia

**Keywords:** Agro-ecological zones, Climate change, Food systems, Governance, Land cover, Land use

## Abstract

We study changes in crop cover under future climate and socio-economic projections. This study is not only organised around the global and regional adaptation or vulnerability to climate change but also includes the influence of projected changes in socio-economic, technological and biophysical drivers, especially regional gross domestic product. The climatic data are obtained from simulations of RCP4.5 and 8.5 by four global circulation models/earth system models from 2000 to 2100. We use Random Forest, an empirical statistical model, to project the future crop cover. Our results show that, at the global scale, increases and decreases in crop cover cancel each other out. Crop cover in the Northern Hemisphere is projected to be impacted more by future climate than the in Southern Hemisphere because of the disparity in the warming rate and precipitation patterns between the two Hemispheres. We found that crop cover in temperate regions is projected to decrease more than in tropical regions. We identified regions of concern and opportunities for climate change adaptation and investment.

**Electronic supplementary material:**

The online version of this article (doi:10.1007/s13280-016-0818-1) contains supplementary material, which is available to authorized users.

## Introduction

The demand for food products has increased in the past 50 years driven by an increasing population and changing dietary choices (Miller 2008). Along with increasing demand has come increasing food insecurity, concentrated in the poorest countries. Food insecurity is the absence of continuing, guaranteed access to adequate nutrition. As defined by the FAO, it has several dimensions: availability, access, utilisation and stability (FAO et al. 2015). A shortfall in the actual amount of food available is referred to as the nutrition gap while failure to get this food to hungry mouths constitutes the distribution gap. Despite continuing high levels of food insecurity, since the mid 1970s the rate of conversion of land for agriculture has slowed (Alston et al. 2009). This could be linked, inter alia, to broad adoption of improvements in agro-technologies, which have led to higher yields (Munns et al. 2012; Hu and Xiong 2014; Pallotta et al. 2014). However, recent projections estimate a median human population of 9.3 billion by 2050 (UN 2012). As a result, actual food supplies must increase by 70 % to meet the demands of population and dietary changes (Bruinsma 2003). Even with significant attention to the components of the distribution gap, existing agricultural systems will be under pressure to meet this demand, implying a need for either continuing increases in yields or increased areas under agriculture, or more likely, a combination of both. In this study we focus on changes in the area under cultivation.

Existing evidence suggests that future climates may have a negative impact on global food production (Easterling and Apps 2005; Battisti and Naylor 2009). For example, Lobell et al. (2008) evaluated changes in crop yields and assessed agricultural vulnerability to climate change, identifying regions that without adaptation will be at risk by 2030, in as much as yields of major crops are expected to decrease there. However, empirical models based on gridded databases such as those used by Lobell et al. (2008), although good at assessing general trends in production, do a poor job of simulating the yield potential of major crops (see for example Lobell et al. 2011; van Wart et al. 2013; Cai et al. 2015a). More specific models parameterised based on the plant physiology, like global gridded crop models (GGCMs) (Rosenzweig et al. 2014) are better tools for determining yield potentials. Current crop growth simulation models [see for example models used by Rötter et al. (2012)] are highly parameterized and some rely on remotely sensed vegetation indices that are used as surrogates for crop yields or cropping area (Prasad et al. 2006; Mussatto et al. 2010; Hu and Xiong 2014). Therefore, forecasting crop cover areas at the global scale is an important step in the modelling process.

Given a certain level of demand, climate and the biophysical attributes of the land are important determinants of crop cover pattern. There is substantial evidence that climate change will impact agricultural systems by changing productivity patterns, with effects ranging from reduced yields in low latitudes to increased productivity at high latitudes but with substantial regional variation (Liang et al. 2011; van Wart et al. 2013; Rosenzweig et al. 2014; Cai et al. 2015a). However, socio-economic drivers are important too. These are generally linked to technology and infrastructure, and to policy and institutional settings. Despite this, current approaches to modelling climate change impacts on agriculture, usually by using statistical approaches (Lobell et al. 2011; van Wart et al. 2013; Cai et al. 2015a), do not account for the impacts of technology or infrastructure. Our default assumption in the modelling described below is that regions with stronger economies are more likely to invest in agro-technologies to enhance agricultural systems or to ameliorate climate change impacts. For example, regions with robust economies are already expanding irrigation schemes in order to minimise risks (ABARES 2010; Mehta et al. 2013; Kidd et al. 2014). It follows that introducing socio-economic drivers into empirical models that already account for climatic and biophysical variables should provide better projections of future crop cover patterns.

We develop an empirical statistical global model based on the present climate, socio-economic, technological and biophysical drivers to identify areas where the existing patterns of crop cover suggest a high sensitivity to climate. This model is then used to project crop cover under future climates based on four global circulation models (GCMs) and two representative concentration pathways of radiative forcing by greenhouse gases (RCP) (Vuuren et al. 2011). A novelty of this study is that we combine agronomic and climatic factors into a compound variable known as agro-ecological zones (AEZ) (Ramankutty and Foley 1999) and use this as an independent variable in the model. We also assume that the geographic locations of the AEZs will be affected by the future climate, so we used projected AEZs based on the GCMs. In short, changing climate will affect the geographic distribution of AEZs and this will have an impact on crop cover.

The ultimate purpose of this exercise is to address the following questions: Will the future climate have a neutral, negative or positive (no change/decrease/increase) impact on crop cover? What will be the pattern of change? How well do the projections of crop cover based on the different GCMs agree? And, is it possible to identify regions of concern and opportunities for climate change adaptation? The output from this model is an essential input to our global integrated assessment models (GIAM) (Newth 2011; Scealy et al. 2012), which combines economic and biophysical descriptions of global development.

## Materials and methods

### Random forest in regression analysis

We have developed our empirical model using the Random Forest (RF) methodology (Breiman 2001). RF is a statistical approach based on the generation of an ensemble of decision trees and can be used for classification or regression analysis. The RF regression analysis is characterised by four steps. First we subset 2/3 of the data by selecting a bootstrap sample. Second for each data bootstrap sample, the variables are also bootstrapped (i.e., only a subset of the variables is considered at each potential split, so a set of potential trees are built). Third, we run an evaluation of the model using a test set data, i.e., the 1/3 of the data that were not selected in the bootstrap sample (step 1), these are called *out*-*of*-*bag* (OOB) observations. Fourth, we build a large number of trees that are averaged (in regression analysis) in order to get a model prediction.

Steps of the procedure:

1. Obtain a bootstrap sample from the dataset.
2. Train a decision tree and constructs a binary tree minimising the error in each tree.
3. Measure OOB errors.
4. As a result of this procedure, we have a large number of trees that are relatively independent that are used to classify a data point by majority of vote among them (in classification analysis) or averaged in order to obtain a data prediction (in regression analysis).

### Bagging and *out***-***of***-***bag* estimation

A way to reduce variance and increase accuracy in statistical learning methods is to take many training sets from the population, to build separate prediction models, and to average the results (James et al. 2013). For example, we can get $\hat{f}^{1} \left( x \right),  \hat{f}^{2} \left( x \right), \ldots ,  \hat{f}^{B} \left( x \right)$ using *B* separate training sets and averaging them to obtain a low-variance statistical model given by

$$ \hat{f}_{\text{avg}} \left( x \right) = \frac{ 1}{ B}\mathop \sum \limits_{b = 1}^{B}  \hat{f}^{b}  (x). $$

However, most commonly, we do not have access to multiple training sets, so an alternative is to bootstrap the original data. Bagging or bootstrapped data sample can be done to reduce the variance of a statistical learning method (Breiman 1996; Liang et al. 2011). In the RF approach, we generate *B* different bootstrapped training data sets that are then averaged to obtain a single low-variance model prediction (James et al. 2013). We then train our model on the *b*th bootstrapped training set in order to get $\hat{f}^{*b} (x)$, and finally average all the predictions, to obtain a regression (bagged) tree:

$$ \hat{f}_{\text{bag}} \left( x \right) = \frac{ 1}{ B}\mathop \sum \limits_{b = 1}^{B}  \hat{f}^{*b}  (x). $$

Each regression tree uses 2/3’s of the observations to train the model, the remaining one-third of the observations (i.e., not used to fit a given bagged tree) are called OOB observations. The OOB observations are used to estimate model accuracy and also provide a measure of strength, correlation between trees and variable importance.

### Training the model: Experimental methodology

The RF methodology avoids the common problem of overfitting that can occur with decision tree approaches to regression. In addition, RF is statistically robust with respect to noise (Breiman 2001). It performs better than most approaches where the relationship between the ‘response’ and ‘explanatory’ variables is strongly nonlinear (Williamson et al. 2014). To ensure computational efficiency, our model ran 1000 trees (models) each run using a random subset of 20 000 grid cells, comprising around 30 % of the global land surface. The 20 000 grid cells used in the RF iterations were randomly selected. Each RF iteration (*n*/1000) used 70 % of the data to train the model, and 30 % to test it. We ran the models in the R package ‘randomForest’ (Liaw and Wiener 2002, 2008), using R Statistical Software (R Development Core Team 2011).

We trained the RF model on the global data set of observed crop cover pattern for the period 1969–1999 provided by Ramankutty and Foley (1999). Ramankutty and Foley used remotely sensed data to derive geographically explicit changes in crop cover. The RF model response variable was the average (from 1969 to 1999) fraction of a pixel covered by crop cover. We used 8 explanatory variables that represented socio**-**economic as well as biophysical and climatic drivers of crop cover (Table 1). Maps of each explanatory variable are shown in Fig. S1 in the supplementary material. The datasets used in the model were disaggregated and resampled to match the resolution and extent of the global cropland cover data, 0.5° × 0.5° grid cell using the ‘Raster’ package in R (Hijmans and van Etten 2010).

**Table 1:** Type, list, and description of the variables used in Random Forest

Type of variable	Variable name	Description	Reference
Response	Global cropland cover	Global cropland data, the fraction of a 0.5 × 0.5 grid cell pixel (~5 km) covered by crops, for the period 1969–1999 was averaged and used as response variable	Ramankutty and Foley (1999)
Climate*	Mean annual temperature and mean annual precipitation	Long-term annual means for temperature and precipitation were calculated for the baseline period (1969–1999), data were obtained from the Climatic Research Unit (CRU). Units: Celsius degrees. Future climate projections for temperature and precipitation were obtained from four Global Climate Models for the periods 2020, 2050 and 2080. These results were sampled onto a 0.5 × 0.5 degree grid according by simple lat-lon position (i.e., no interpolation).Units: mm year^−1^	Jones and Harris (2008)
			Harman et al. (unpublished)
	Agro-ecological zones	The map of global agro-ecological-zones provides a standardised framework for the characterization of climate and terrain conditions relevant to agricultural production	Harman et al. (unpublished)
Socio–economic	Regional gross domestic product	We calibrated regional GDP based on labour and population for 18 regions for the world. Normalized GDP is used here as a proxy for technology and infrastructure at the regional level. Unit–less	Cai et al. (2015b)
Technology	Nitrogen and phosphorus Fertiliser Application	Global fertilizer and manure dataset v1 for the period 1994–1999 obtained from the Socioeconomic Data and Applications Center (SEDAC). Units: Kg/ha	Potter and Ramankutty (2010)
Biophysical	Dominant soils	Harmonized world soil database v1.2 obtained from the International Institute for applied systems analysis (IIASA)	Fischer and Nachtergaele (2008)
	Elevation	Elevation layer. Units: metres	Leemans and Cramer (1991)

* The GCMs used in this study are: ACCESS1.3 (ECS = 3.54 K, TCR = 1.64 K) (Dix et al. 2013), CanESM2 (ECS = 3.69 K, TCR = 2.4 K), IPSL_CM5A_LR (ECS = 4.13 K, TCR = 2.0 K) and MIROC5 (ECS = 2.72 K, TCR = 1.5 K) (Forster et al. 2013)

### Data

In the following sections, we discuss each of these explanatory variables. Climate and biophysical variables constrain areas where crops can be successfully grown. This is because temperature and precipitation impose physiological thresholds on plant growth. Biophysical variables, such as soil type, provide different opportunities for the cultivation of a variety of crops through, for example, different nutrient contents or soil texture. Elevation is another biophysical variable that relates with temperature gradients, rain regime and edaphic characteristics (Leifeld et al. 2005). The combination of these uncorrelated climatic and biophysical variables provides a good overall picture of the current suitability of land for agriculture. The AEZs data, although they may be strongly dependent on temperature and precipitation, combine these climatic data with other inputs to provide a compound variable that specifically reflects agronomic factors, like growing degree days and the length of the growing period. A detail explanation of AEZ and the parameters used to calculate the projected AEZ (hereafter PAEZs) is given in the supplementary material. Finally, the socio-economic and technological variables, gross domestic product (GDP) and fertiliser application respectively, add another important dimension to the analysis that represents a region’s capacity to achieve good agricultural outcomes.

## Results

### Simulation of baseline conditions

**Fig. 1:**
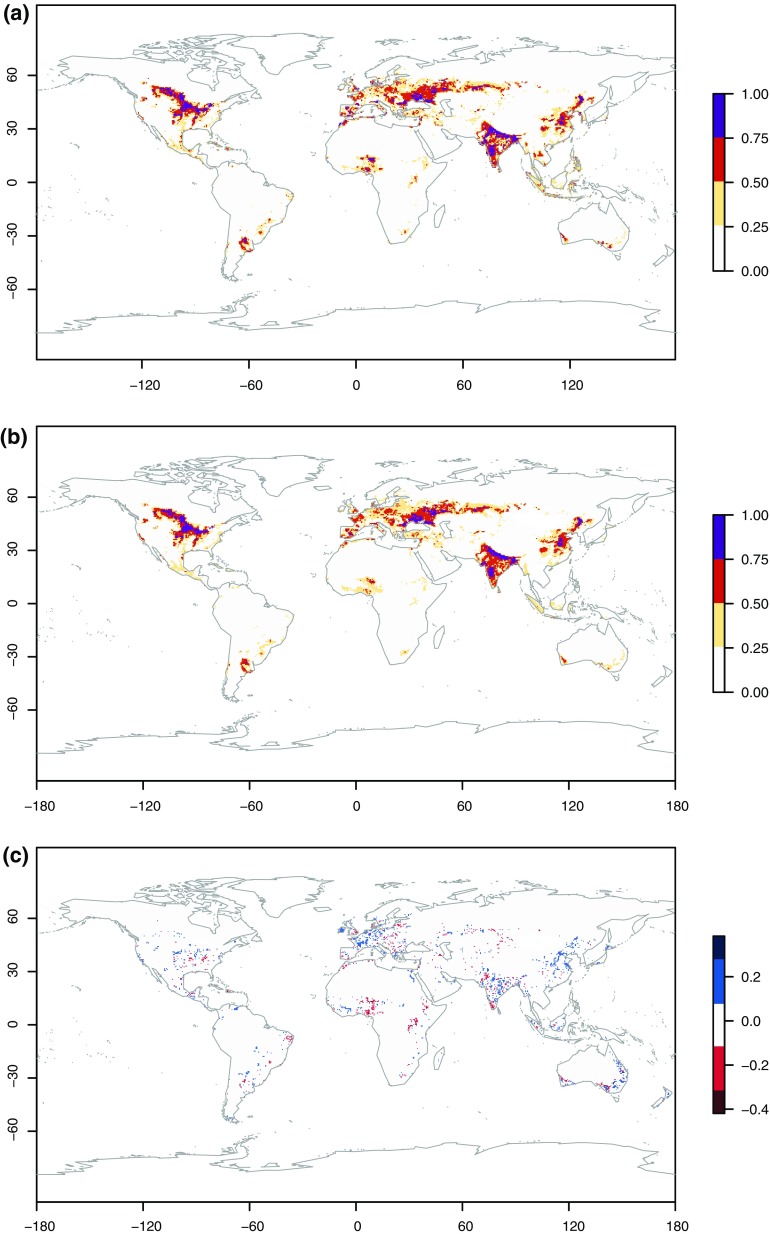
**a** Map of current crop cover and **b** random forest realisation of crop cover for the baseline period, used for cross-validation and **c** the absolute difference between **b** and **a**

We compared the RF model output for the baseline period 1969–1999 to the observations of Ramankutty and Foley (1999). The Random Forest projection for the baseline period explained 92 % of the variance with an average mean squared error of 0.0025 (units = proportion of a grid cell covered by crop (%)). The cross-validation for the realisation of crop cover for the baseline periods, using in each RF iteration 30 % of the observations (i.e., data that were randomly excluded from the model training and only used for testing the results), showed good agreement *R* ^2^ = 0.96 (*P* < 0.0001). There was also good spatial agreement between the Random Forest model realisation and Ramankutty’s and Foley’s (1999) data, although the model tended to exaggerate increases in cover around regions with small fractions of crop cover (Fig. 1).

Our baseline results reconstruct the major global agricultural patterns observed by Ramankutty and Foley (1999). Agricultural regions in the central and north-east USA, Australia, Europe, India, China and the Argentinean Pampas have been reproduced within accuracy (±10 %). In general, our model overestimates crop cover in central Europe, east India and China by about 20 %. By contrast, the model underestimates crop cover in Nigeria by almost 40 %, and this is the largest discrepancy between our model and the observed data. To a lesser degree, the model underestimates crop cover in smaller patches scattered in Africa, Europe and the rest of the world by less than 20 %.

These discrepancies can be linked in large part to the influence of regionally aggregated explanatory variables. The two most important variables in the model are dominant soils, followed by regional GDP (Table 2). These two variables are aggregated by region and our results highlight some trade-offs of using regionalised data. For example, Nigeria is the country with highest GDP in the north-west Africa region, so the aggregated GDP for that region will disadvantage Nigeria by combining it with neighbouring countries with smaller GDP. We tested our model by using non-aggregated GDP as a variable, but the results were less accurate overall because of other disparities such as those between the GDP of countries like Monaco with large GDP but zero crop cover. So by using regionalised variables, we trade-off some detailed information about the magnitude of crop cover to gain a more accurate global spatial pattern that reflects the baseline conditions better.

**Table 2:** Ranking of explanatory variables according to their importance as measured by random forest. The importance measures show how much MSE increases when a variable is randomly permuted. The greater the effect of the variable to reduce MSE, the higher the variable is ranked

Variable	%IncMSE
Dominant soils	127.3
Regional gross domestic product	102.7
Altitude	98.5
Nitrogen fertiliser application	88.0
GAEZ	80.0
Mean annual temperature	72.7
Mean annual precipitation	70.7
Phosphorus fertiliser application	69.3

### Simulations under future climates

We calculated the difference between the projected and the baseline crop cover for all GCMs and RCPs. We present this information in two ways: (1) the direction of change: does the future crop cover increase or decrease relative to the baseline; and (2) the magnitude of change: are the projections, for example, 5, 30 or 50 % higher or lower than the crop cover of the baseline period. In presenting the direction of change, the projections from the four GCMs were compared to obtain maps of total or partial agreement for the two RCPs. In the following section, we show agreement maps that rank from 0, meaning areas projected to be unsuitable for agriculture in all model projections, to 4, where the RF projections based on the four GCMs all agree about the direction of change, that is, an increase or decrease in crop cover. The magnitude of change for each GCM/RCP projections is presented independently.

#### Direction and magnitudes of change

The RF model projects that crop cover will increase in over 75 % of grid cells by the period 2070–2100 (Table 3). However, in the majority of these grid cells the magnitudes of change are small and they are located in regions that have currently very little crop cover. For example, a pixel with 1 % crop cover that is projected to increase to 1.002 % in the future will be registered as a positive change. If we concentrate only on cells where crop cover is projected to change by, for example, more than 5 % then the projected changes are substantially more conservative.

**Table 3:** Direction of change in crop cover relative to the total number of grid cells with crop cover values greater than zero in the baseline period. Grid cells with NoData values were not taken into account to calculate the proportion of change (see Fig S3)

GCM	RCP	Proportion of grid cells with higher crop cover than baseline	Proportion of grid cells with smaller crop cover than baseline	Proportion of grid cells that do not change
ACCESS1.3	4.5	0.784	0.193	0.023
CanESM2	4.5	0.782	0.195	0.022
IPSL_CM5A_LR	4.5	0.782	0.195	0.024
MIROC5	4.5	0.778	0.200	0.023
ACCESS1.3	8.5	0.789	0.191	0.020
CanESM2	8.5	0.791	0.189	0.020
IPSL_CM5A_LR	8.5	0.789	0.189	0.022
MIROC5	8.5	0.785	0.195	0.020

There are areas where the projections indicate a transition from zero to non-zero crop cover in the future (see Fig S3 in the supplementary material). We refer to these areas as ‘novel’. Most of the novel crop cover is found in arid and/or boreal regions currently marginal for traditional agriculture. For example, in central Australia, and tropical regions in Africa and Latin America the models project an increase in crop cover of about 20 % (see Fig. S4 in the supplementary material). In the case of central Australia, the biggest limitations for traditional agriculture are imposed by soil quality and climate conditions. In central Africa and Latin America, in addition to some potential biophysical limitations imposed by soil quality, there are political and/or institutional drivers that prevent the establishment of cropping systems. Although GDP is a good socio-economic descriptor, some social and institutional realities cannot be well described by this indicator and are therefore not captured by our modelling system. Consequently, these novel regions should be treated with caution.

An intercomparison between the two RCP projections, across the four GCMs, shows a higher level of agreement between individual model projections for RCP 8.5 than RCP 4.5. In the context of this analysis, temperature is a key variable for calculating the AEZs and PAEZs because this variable has a direct impact on the temperature threshold above which crops can grow. Although we do not use temperature thresholds as explanatory variables in the RF model, they are indirectly considered by using the AEZ and PAEZs. The GCMs we selected cover a wide cross section of equilibrium climate sensitivities (ECS), which are a large contributor to differences between models (Dix et al. 2013; Forster et al. 2013). That is, for a given radiative forcing, represented by the RCP scenarios, the different GCMs will reach a given level of global warming more or less rapidly. The good level of agreement we see between the RCP 8.5 model projections relates to the fact that all four GCMs reach about +2 °C of global warming by 2050 (Knutti and Sedláček 2012). For example, the transient climate response (TCR) in ACCESS 1.3 is 1.64 K. An analysis of 23 CMIP5 models found a similar range of 1.1–2.5 K with median 1.8 K (Forster et al. 2013).

**Fig. 2:**
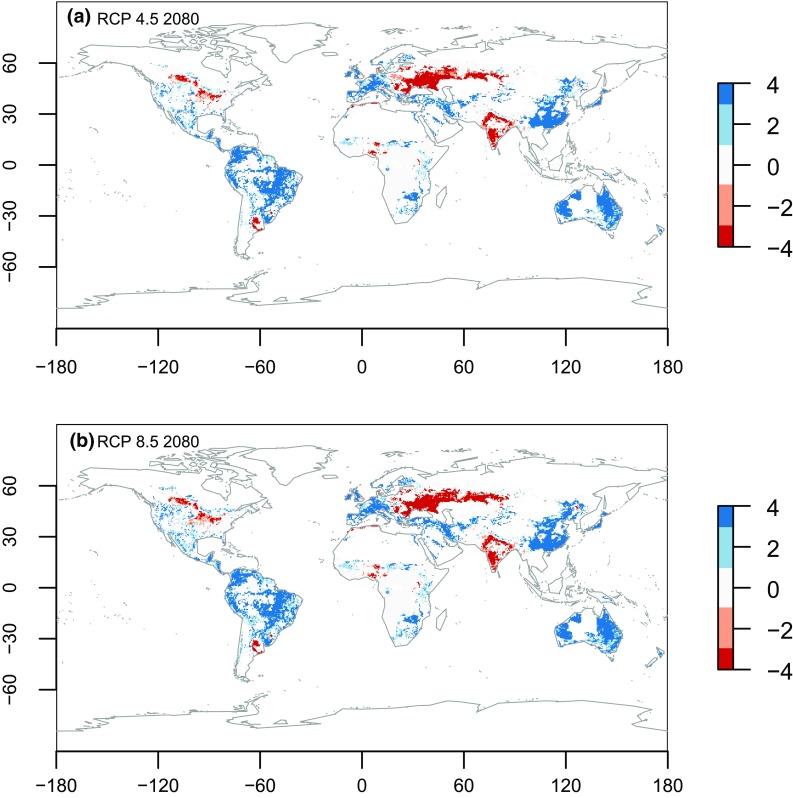
Maps of total or partial agreement on the direction of change (increase or decline) in the crop cover by 2080. **a** shows the model agreement between the 4 GCMs for the RCP 4.5; **b** shows model agreement between the 4 GCMs for the RCP 8.5. Values of 0 = no agreement, 1–4 = one to four models agree that the direction of change will be positive; from −4 to −1 = where one to four models agree the direction of change will be negative

**Fig. 3:**
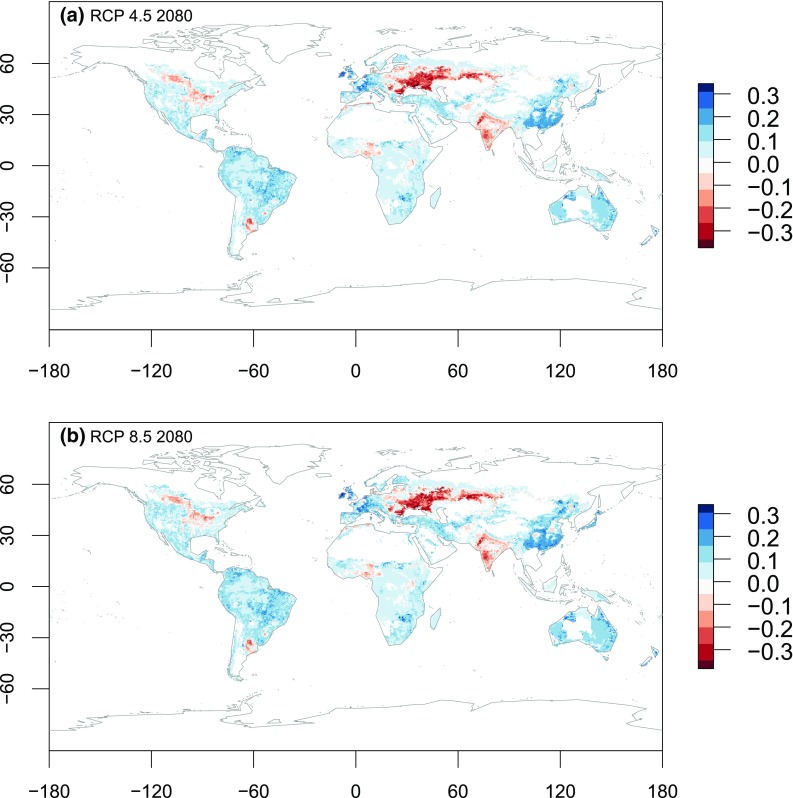
Magnitudes of change in the projected crop cover relative to the baseline period. Positive values (*blue*) indicate a projected expansion in crop cover; negative values (*red*) a projected contraction in crop cover. Magnitudes of change are presented as a proportion of existing crop cover. The magnitudes presented in these maps are ensembles of the 4 GCMs. The magnitudes of change for each model can be found in Figs. S5 and S6 in the supplementary material

Our results suggest that large areas of agricultural production in the Northern Hemisphere may become vulnerable to climate change, with a marked decrease in crop cover. The maps shown in Fig. 2 only reflect the direction of change (increase/decrease) and do not reveal the magnitude of change. As noted above, the direction of change in the projected crop cover provides some insight of the impact from climate and socio-economic change but this information alone is incomplete. The magnitude of change is shown in Fig. 3 and reveals that the steppes region in Eastern Europe will display the greatest decrease in crop cover, reducing by around 30–40 %. This is mostly due to a shift in the AEZs geographic pattern. West India, the Argentinean Pampas and north-west USA may decrease their crop cover also by around 10–20 %. Nigeria is projected to decrease its crop cover by around 10 %. A large increase in crop cover is projected for East Asia, and Europe, and a smaller increase in Australia. The crop area increase in Australia is both large and novel, so it may have a relatively large impact in terms of quantity of output. South America, USA, South and Central Africa and boreal regions in general all show increases of about 10 % (Fig. 3). It is also important to note that model errors averaged at 0.0025 % of crops cover, which is a small so gives the model high levels of certainty.

## Discussion

Averaged over the global land mass, increases and decreases in crop cover as a result of climate change tend to cancel each other out. The proportion of land where crop cover is projected to increase is greater than the proportion that is projected to decrease. However, this statement comes with qualifications. First, the magnitude of change is very small in a large number of grid cells, that is the aggregate decrease or increase could be small. Second, we found substantial agreement between projections based on output from the different GCMs and RCPs about the direction and magnitude of change in areas where crop cover would decrease, for example in Eastern Europe. This is of particular concern given that food supplies are needed to increase by 70 % to meet population demands by the mid–end of this century (Bruinsma 2003). The area where crop could potentially grow was not projected to increase significantly. Therefore, the option to meet the 70 % increase in food supplies would depend on agricultural intensification.

Some regions in the Northern Hemisphere will become vulnerable, receiving a marked decrease in crop cover. All GCMs for the two RCPs agreed on the direction of change. This relates to a shift in the geographic pattern of the PAEZ, which is linked to an expansion of the tropical zone, one of the three climate zones (tropical, temperate and boreal) that define the AEZs. The tropical zone, based on the AEZ methodology by Ramankutty and Foley (1999), is defined by the minimum temperature and the growing degree days. Tropical regions are projected to expand, and the minimum temperatures are projected to exceed the threshold that defines this climatic region. However, the magnitude of change varied between the RCPs by up to 20 %. Most discrepancies were found in boreal regions, areas with high elevation and in central Australia, a region characterised by desert, shrub land, temperate grasslands and savannas. We identified regions of concern and opportunities. Regions that may see a moderate increase in crop cover, such as East Africa, Asia and Latin America, where all models agreed on the direction and magnitude of change, should be a priority target for adaptation (Lobell 2014) and investments. This, however, can be constrained by sustainable development goals and policy settings. Our regions of concern align with those of Lobell et al. (2008) who forecast that South Asia and North-West Africa would be vulnerable under future climate. But in addition, we highlight that non-food-insecure regions with a stronger socio-economic structure, like Europe, the Argentinean Pampas, and central USA, may also see a decrease in crop cover in future climates. These regions may invest in new low carbon emission technologies to ameliorate the impacts of climate change on crop cover.

A region’s vulnerability to climate change is a response to a combination of climatic, biophysical, technological and socio-economic drivers. From the climate perspective, some important conclusions can be drawn from these projections. First, changes in crop cover in temperate regions are expected to be larger than in tropical regions and so are affecting regions that currently are food secure, such as some areas in the USA, South America and Europe. This finding reflects climatic findings of Battisti and Naylor (2009): that temperate regions will experience larger year-to-year variations in temperature and precipitation than tropical regions. Second, we found that the Northern Hemisphere is more vulnerable to climate change than the Southern as it sees larger and more severe losses in crop cover. This could be due to a disparity between the warming rates and precipitation patterns in the hemispheres, where the Northern Hemisphere has a faster warming rate and projected decrease in rainfall (Friedman et al. 2013; Li et al. 2013) than the Southern. The disparity in warming rates relates to land mass proportions and global ocean currents that contribute to the warming in the Northern Hemisphere. The results for the 2070–2100 period for RCP 4.5 and RCP 8.5 showed similar spatial patterns but different magnitudes of change, although we found better agreement between models under RCP 8.5 (due to the GCM’s climate sensitivities as explain in "Direction and magnitudes of change" section), as well as a larger number of grid cells projected to change. Third, from the food-security perspective, local studies at finer geographic resolutions are still needed to identify concerns or opportunities within the regions, at landscape and farm scales. Regional studies should be undertaken with, for example, physically driven dynamically downscaled climate data (White et al. 2013) instead of interpolated data.

The socio-economic and technological variables used in this analysis are associated with some caveats. We acknowledge that physiology driven models may yield more accurate outputs, but our objective was to obtain a crop cover projection at a large scale, the global scale, for a long time period. The computational requirement and model-parameterization needed to run specific physiological models was out of the scope of this study. We have already mentioned that aggregated data may be useful to predict the big picture about crop cover, but at the cost of losing detailed information for specific regions. This is the case for Nigeria, which has the highest GDP in its region but, because of the aggregation, the regional GDP is lower than the Nigeria’s, so its crop cover is slightly underestimated. One caveat of the technological indicators we used—areas where fertiliser were applied—is that: (1) they are static over time and (2) the spatial patter clearly denotes political limits, which in some cases could be realistic as different countries or states, as in the case of Australia, may have different fertilisation managements. About the first caveat, fertiliser application being static over time, we acknowledge this limitation in our analysis; however, we emphasise that projecting future fertiliser application at the global scale is a major task (Scott et al. 2002) that was out of the scope of this study. In addition, any modelled map showing future fertiliser application will be subject to uncertainties that will add to those already existing in our model, therefore making our projection difficult to interpret.

## Conclusions

Under the current climatic circumstances and CO_2_ emission trajectories, a shift in crop cover patterns towards the end of the century is likely to occur. Therefore, the option to meet the 70 % increase in food supplies would depend on agricultural intensification and investment in new low and/or negative CO_2_ emission technologies. In the context of this study crops were allowed to expand and grow in places where the socio-economic, climatic and biophysical conditions were optimal. So we assumed that crops are completely mobile. However, our results indicated that crop cover did not expand significantly outside its existing bounds, or at least the novel agricultural systems projected in our models are in marginal land, and the magnitude of change in crop cover in the novel systems is small, no greater than 10 %.

## Electronic supplementary material

Below is the link to the electronic supplementary material.

Supplementary material 1 (PDF 1296 kb)
