# Supplementary material for: Patterns of crop cover under future climates
Source: Ambio. 2016 Sep 23;46(3):265–76. doi: 10.1007/s13280-016-0818-1 (PMC5347521; doi:10.1007/s13280-016-0818-1)
Supplement: Supplementary file 1 — Supplementary material 1 (PDF 1296 kb) [file 13280_2016_818_MOESM1_ESM.pdf]

Patterns of crop cover under future climates

Electronic supplementary material (ESM)

Authors:

Luciana L. Porfirio[1]; David Newth[1]; Ian N. Harman[1]; John J. Finnigan[1]; Yiyong Cai[2]

Affiliations

[1] Oceans & Atmosphere. The Commonwealth Scientific and Industrial Research Organisation (CSIRO). Yarralumla, ACT 2601, Australia

[2] Centre for Applied Macroeconomic Analysis. The Australian National University. JG Crawford Building, 132 Lennox Crossing, Acton ACT 0200, Australia

Corresponding author: Luciana L. Porfirio; E: Luciana.Porfirio@csiro.au

Material and Methods

The RF algorithm contains three major steps: (1) *n* 'bootstrap' samples are drawn from the original training data with each sample comprising 70%, of the data. The remaining 30% of the data are used to estimate model errors and accuracy and later used for cross-validation; (2) an unpruned regression tree is grown for each of the bootstrap samples. However, rather than using the best split among all *p* predictors, only *m* of the *p* predictors are randomly sampled and the best split is chosen from among these *m* variables. And (3), the prediction is formed by averaging the output of the *n* trees. RF also produces scores measuring the relative importance of each explanatory variable on the response variable. This score is estimated by calculating the mean decrease in accuracy due to the permutation of one explanatory variable while leaving the others unchanged (Liaw and Wiener 2002). An overall measure of the mean squared errors (sum of squared residuals divided by number of trees) is also calculated within RF.

Data

Climate based variables

Temperature and Precipitation

We calculated the difference between projected and baseline yearly mean temperatures and precipitation for two RCP scenarios: RCP4.5 and RCP8.5 (van Vuuren et al., 2011), using four GCMs from the CMIP5 archive (Taylor et al. 2012) . The baseline period was 1969-1999 and the projected period was 2071-2100. These changes in temperature and precipitation were used to calculate the climate metrics necessary to compute the future (projected) AEZs (PAEZs). The GCMs we used, with their equilibrium climate sensitivity (ECS) and transient climate response (TCR) were: ACCESS1.3 (ECS= 3.54K, TCR= 1.64K) (Dix et al. 2013), CanESM2 (ECS=3.69K, TCR=2.4K), IPSL_CM5A_LR (ECS= 4.13K,TCR=2K) and MIROC5 (ECS= 2.72K, TCR=1.5K) (Forster et al. 2013). These GCMs are representative of the range of climate sensitivities in the CMIP5 archive. The climate projections from each of the four GCMs were used independently in Random Forest. That is, we did not calculate a climate model ensemble, but ran the RF model for each GCM and each RCP separately.

AEZ

The agro-ecological-zones (AEZs) dataset is widely used in agronomic, economic and integrated assessment models to determine where different crops could be grown. For example, the Global Trade Analysis Project (GTAP) model determines for each economic region an endowment of land in each of the 18 AEZ's. In this study, we used AEZs calculated by Harman (*in prep.)* based on a methodology that reproduces the results of Ramankutty and Foley (1999) for the reference period (1969-1999). Their original method divides the total global land surface into 18 AEZs in a two-step process. First is a partitioning into three climate zones, tropical, temperate and boreal, based on two climate metrics: the minimum over the year of the daily minimum temperature and growing degree days (GDD), a measure of heat accumulation used by agronomist to predict plant growth rates and phenological stages. Second, each climate zone is further sub-divided into 6 land types according to the length of the growing period (LGP). LGP is defined as the period of time in a given year when the climate is optimal for plants to grow and complete a phenological cycle. GDD and LGP are well-established metrics from agronomy research though it should be recognised that there is an inherent scale issue between the

plot-scale research that underpins their definition and their use in global scale climate models and economic models.

Although an AEZ map developed by Ramankutty and Foley (1999), which input data have been reproduced by IIASA and FAO and known as GAEZ (v3) (Fischer and Nachtergaele 2008) is already in relatively wide use, the GAEZ data set cannot be extrapolated to account for climates different to that of its baseline period, 1969-1999. Hence we have used a generic, objective and replicable methodology to reconstruct the AEZs. This methodology accommodates changes in the climatic factors that are determinants of the AEZs (Harman *in prep.).* The defining climate metrics and the resultant AEZ distribution for the GAEZ 1969-1999 base period have been reconstructed using readily obtainable, curated and assured datasets, but it was necessary to modify some aspects of the definitions of GDD and LGP to be numerically more robust. Our baseline AEZs are based on the gridded, re-analysed, monthly-averaged, daily minimum and near-surface air temperature from the Climate Research Unit CRU-TS3.21 data (Jones and Harris 2008). Differences between GTAP-AEZ and Harman's (*in prep*) can be seen in around 2.8% of grid cells along the tropical-temperate climate zone boundary and 2.7% of grid cells along the temperate-boreal climate zone boundary, where cells are classified into different AEZs by the two approaches. These discrepancies are due to the different baselines used in the generation of the AEZs. To extend our crop cover model projections to future times with changing climates, projected AEZs (PAEZs) were then constructed based on projected climate metrics. These projected metrics (daily minimum temperatures, GDD and LGP) were generated using climate data from four Global Climate Models (GCMs) sourced from the Coupled Model Intercomparison Project Phase 5 (CMIP5) database as described in 2.2.1.

Biophysical variables

Terrain elevation and dominant soil maps were used both as direct explanatory variables in the Random Forest model and also as inputs to the AEZs and PAEZs. These variables were assumed to be unchanged between the baseline and future periods.

Socio-economic variables

GDP

We use a set of two normalised (ranking from 0 to 1) GDP projections that corresponds to the two RCPs, 4.5 and 8.5 (Cai *et al.* 2015). These GDP projections were generated using GTEM-C, the CSIRO variant of the Global Trade and Environment model (Cai et al. 2015). In the first scenario, global population was assumed to follow the median variant of the United Nations World Population Projections, reaching 10.6 billion people in 2100 (UN 2012). The supply of fossil fuels was assumed to continue the current trend of growth while assumptions about industrial and household energy efficiency improvements were conservative, ranging from 0.15% to 0.75% per year Cai *et al.* (2015). As such, global greenhouse gas (GHG) emissions followed RCP8.5, reaching 130 gigatonnes $GtCO_2$ in 2100. Based on the above assumptions, regional total factor productivities (TFP) were iteratively derived such that regional Gross Domestic Products (GDP) will continue the momentum of growth, with world GDP reaching 450 trillion in constant 2007 US dollars (2007 US$). The second scenario adopted the same assumptions about demographic change, energy efficiency improvement and regional TFP, but imposed a (uniform) global carbon price, to ensure that global GHG emissions followed RCP4.5. In this

scenario, the economy responded by switching away from energy and carbon intensive industrial practice and consumption patterns. However, the overall differences in GDP between the two scenarios were minor. This is because the economic cost of decarbonisation was contained by the uptake of renewable energy generation and carbon sequestration technologies in the short term and offset by the avoidance of climate damages in the long term. As a result, the ranking of GDP by region did not change (Fig. A2 in Supplementary information file), although there were differences in the actual monetary values (for further details, see Cai *et al.* 2015).

Technology

Nitrogen and phosphorus Fertiliser Application data were sourced from the Global Fertilizer and Manure dataset v1 for the period 1994-1999 obtained from the Socioeconomic Data and Applications Center (SEDAC). Units: Kg/Ha (Potter and Ramankutty, 2010).

See Partial Dependence Plot foo all variables (Figure S7).

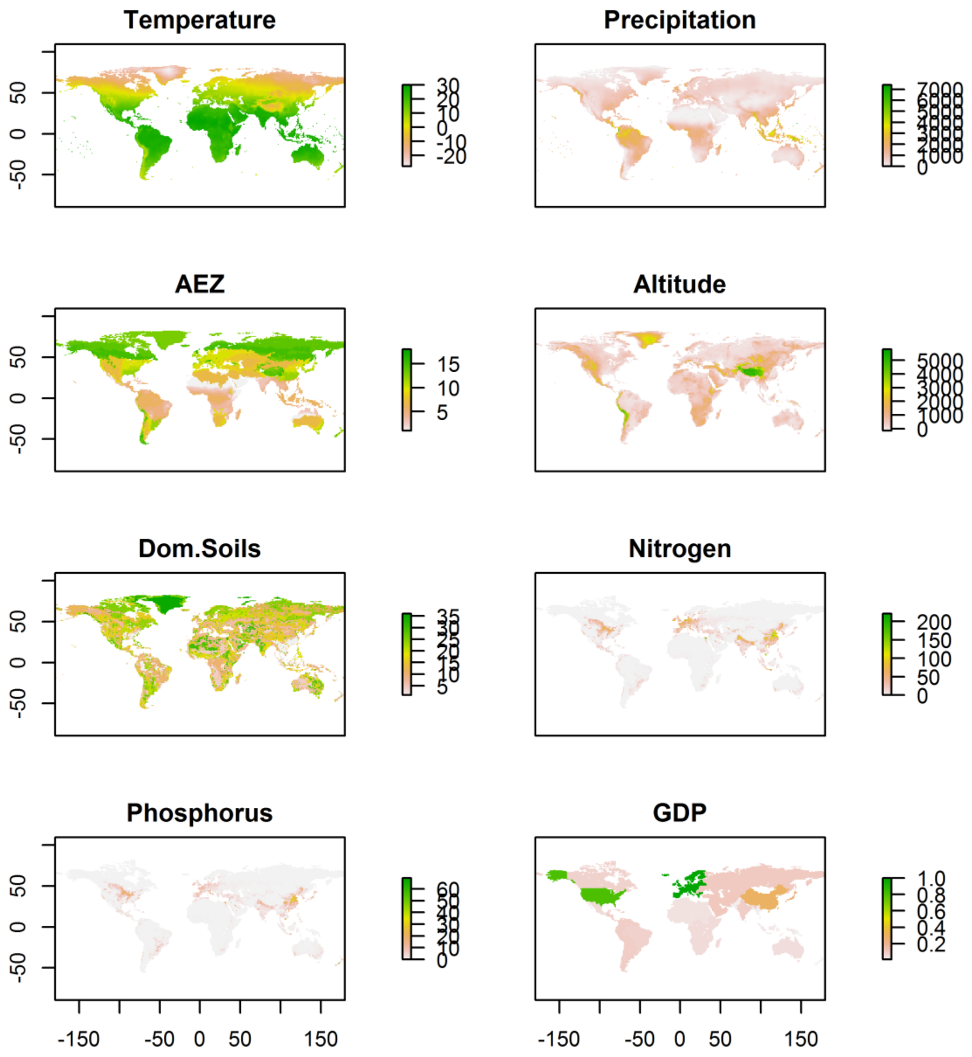

Fig S1. Maps of covariates used in Random Forest. A full description, references and the units for each covariate can be found in Table 1.

Table S1. Regional aggregation in GTEM-C model

Code	***Name***	***Regions***
USA	USA	United States of America
EUR	Europe	Albania, Austria, Belarus, Belgium, Bulgaria, Canada, Croatia, Cyprus, Czech Republic, Denmark, Estonia, Finland, France, Germany, Greece, Hungary, Ireland, Italy, Latvia, Lithuania, Luxembourg, Malta, Netherlands, Norway, Poland, Portugal, Rest of EFTA, Romania, Slovakia, Slovenia, Spain, Sweden, Switzerland, United Kingdom
CHN	China	China, Hong Kong
JPN	Japan	Japan
IND	India	India
BRZ	Brazil	Brazil
CAN	Canada	Canada
AUS	Australia	Australia
NZL	New Zealand	New Zealand
IDN	Indonesia	Indonesia
MEX	Mexico	Mexico and Rest of North America
RLA	Rest of Latin America	Argentina, Bolivia, Caribbean, Chile, Colombia, Costa Rica, Ecuador, El Salvador, Georgia, Guatemala, Honduras, Nicaragua, Panama, Paraguay, Peru, Uruguay, Venezuela, Rest of Central America, Rest of South America
RSA	Rest of South Asia	Bangladesh, Pakistan, Sri Lanka, Rest of South Asia
RNEA	Rest of Northeast Asia	Korea, Lao People's Democratic Republic, Malaysia, Mongolia, Nepal, Philippines, Singapore, Taiwan, Thailand, Viet Nam, Rest of ASEAN, Rest of East Asia, Rest of Oceania
FSU	Former Soviet Union	Armenia, Azerbaijan, Georgia, Kazakhstan, Kyrgyzstan, Russian Federation, Ukraine, Rest of Europe
NAF	Northern Africa	Egypt, Morocco, Tunisia, Rest of North Africa
SAF	Southern Africa	Botswana, Cameroon, Cote d'Ivoire, Ethiopia, Ghana, Kenya, Madagascar, Malawi, Mauritius, Mozambique, Namibia, Nigeria, Senegal, South Africa, Tanzania, Uganda, Zambia, Zimbabwe, Central Africa, South Central Africa, Rest of Western Africa, Rest of Eastern Africa, Rest of South African Customs
MDE	Middle East	Bahrain, Islamic Republic of Iran, Israel, Kazakhstan, Kuwait, Oman, Qatar, Saudi Arabia, Turkey, United Arab Emirates, Rest of West Asia

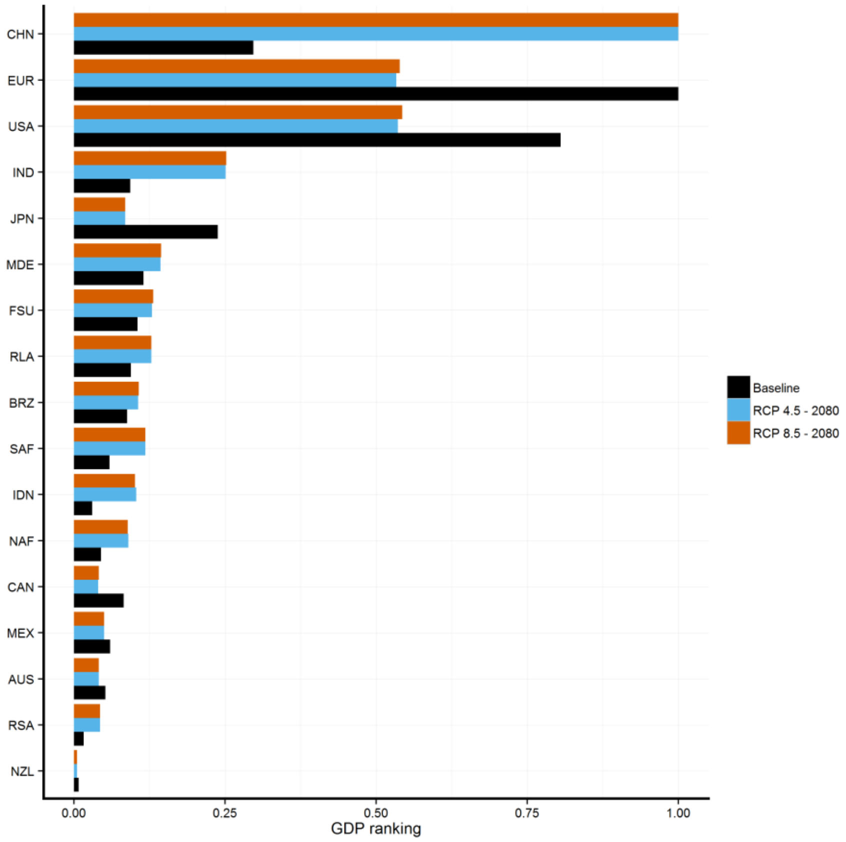

Figure S2. GDP estimates for the baseline periods and projections to 2080 based on the RCPs 4.5 and 8.5. Source: projections obtained from the GTEM-C model (Cai *et al.*, 2015).

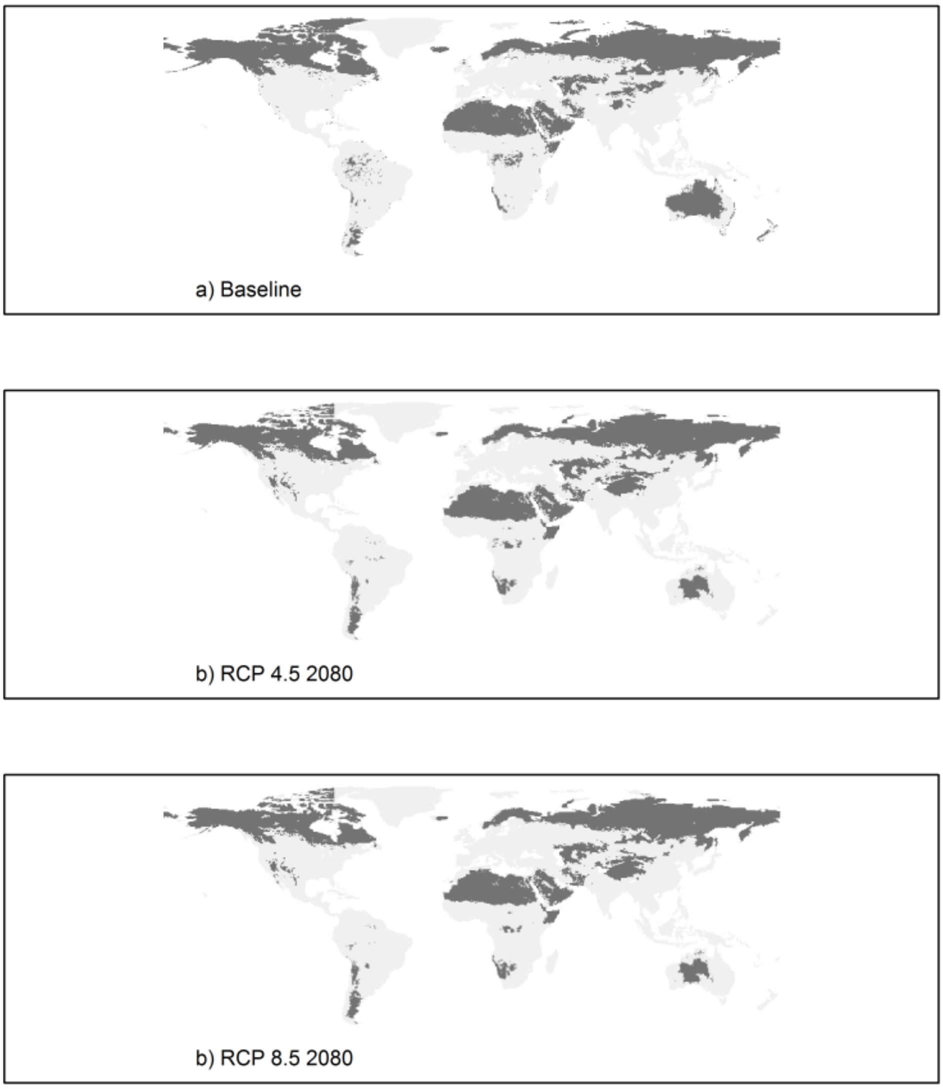

Figure S3. Zero crop cover in the baseline and projected in the future predictions. Each map shows any pixel that was zero in the baseline period and its crop cover was projected to have very small value, e.g. smaller than 0.05%.

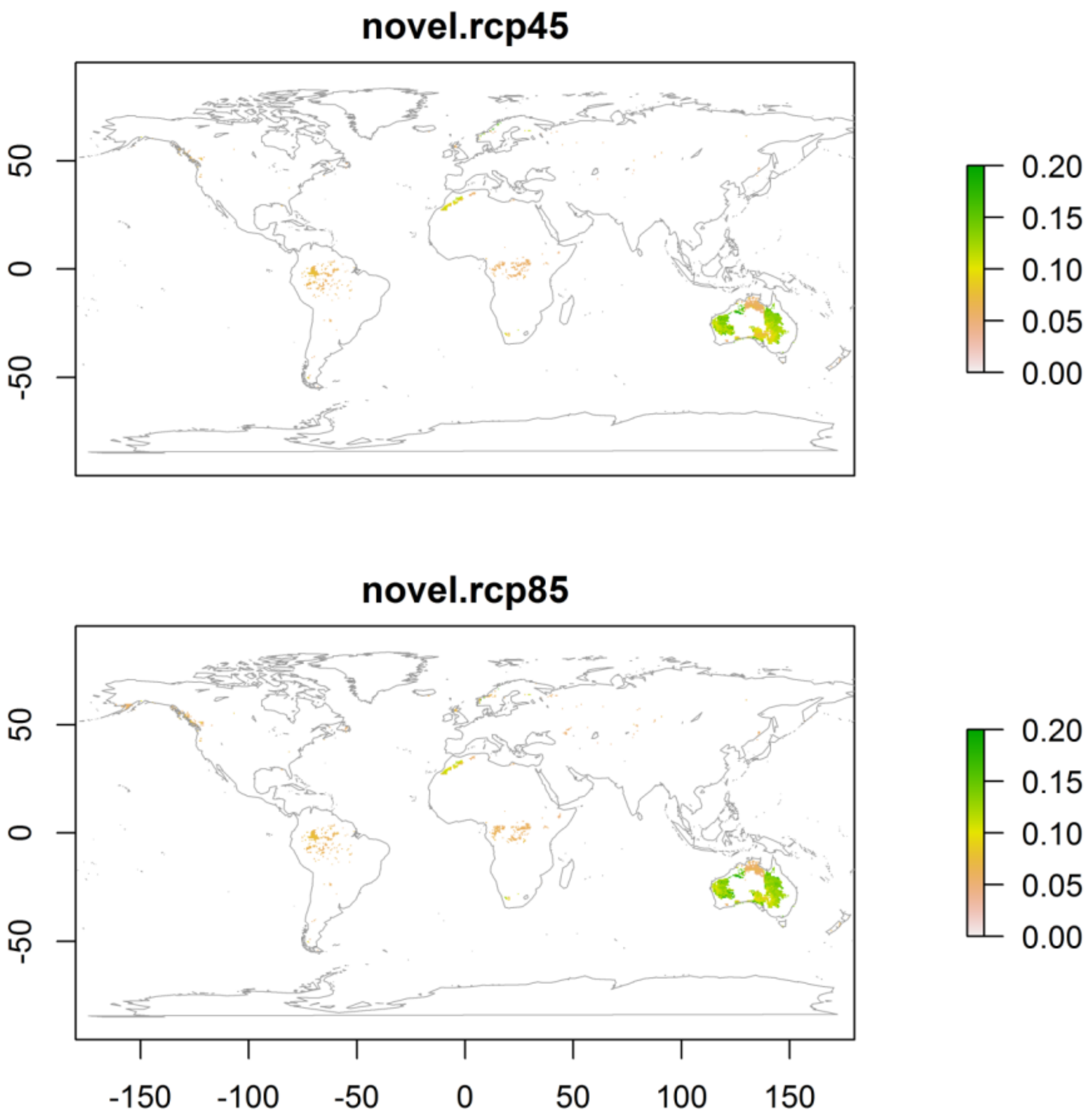

Figure S4. Ensemble mean of projected novel agricultural systems. The maps show areas that present zero crop cover in the baseline period increase to greater than 10% cover in the projections by the 2070-2100 period. Each pixel shows the average value for the 4 GCM, the two maps show ensembles of projected agricultural systems for the 2 RCPs.

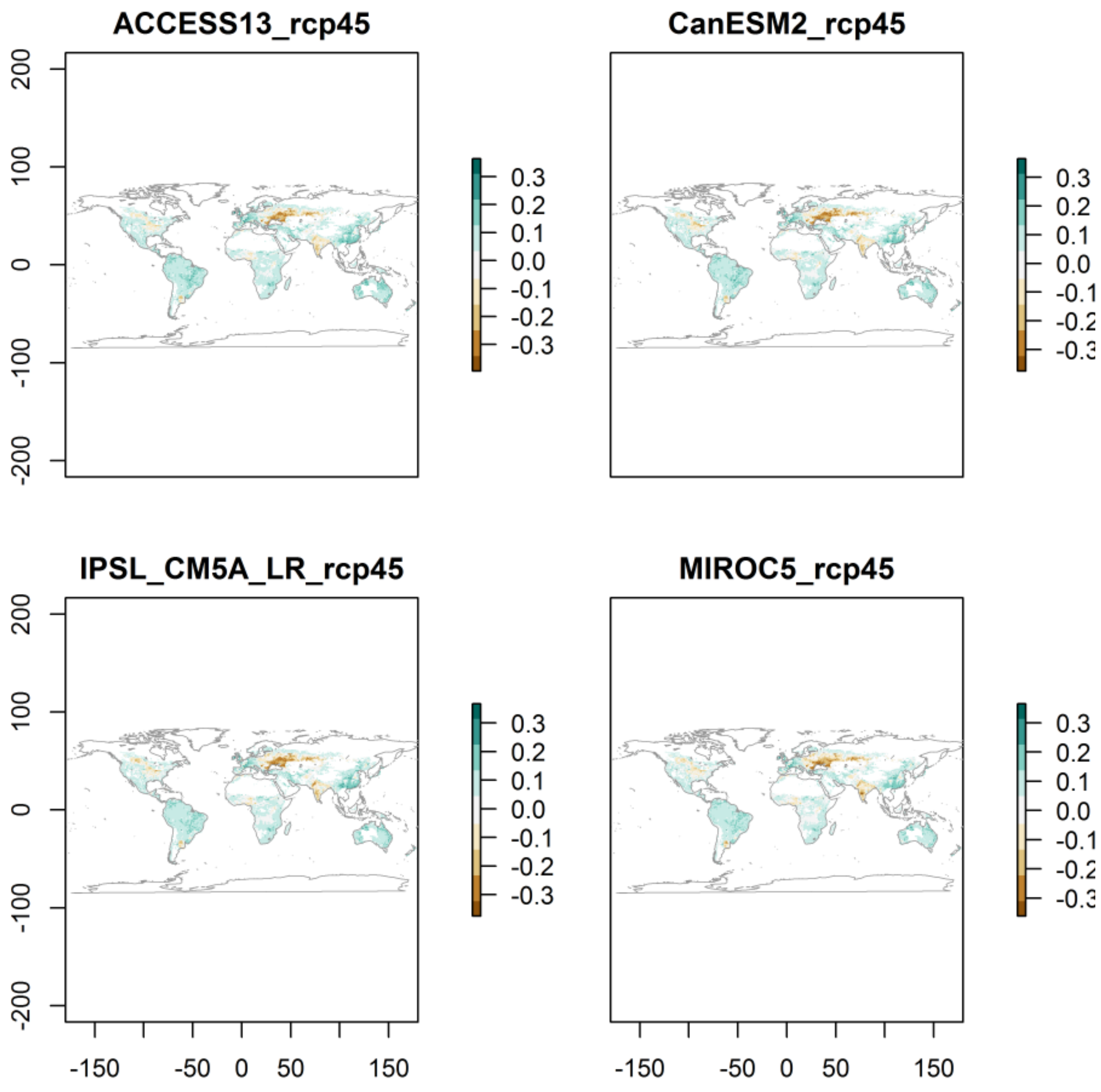

Figure S5. Magnitude of change in the projected crop cover using information from the 4GCMs under the RCP4.5 scenario.

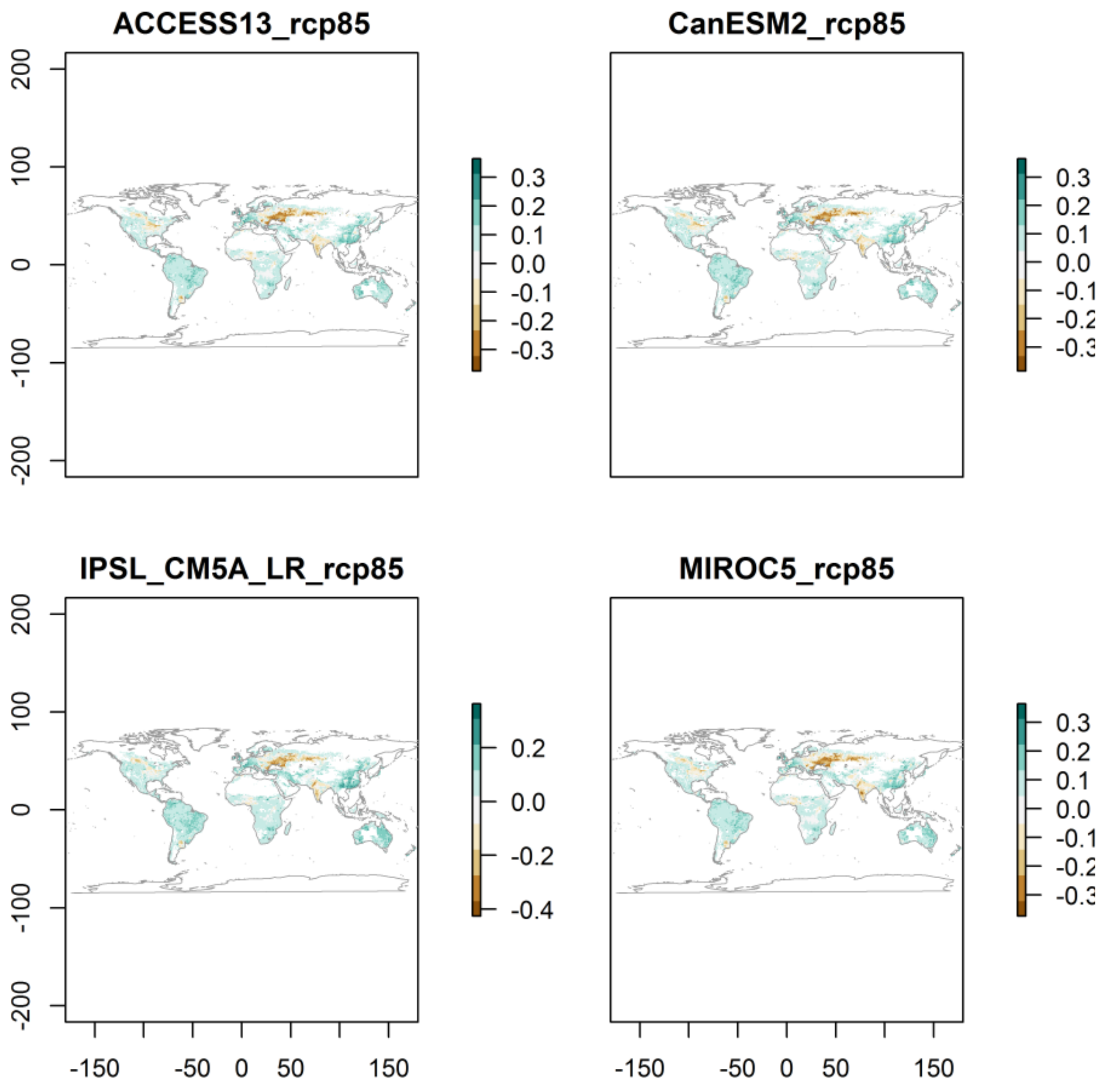

Figure S6. Magnitude of change in the projected crop cover using information from the 4GCMs under the RCP8.5 scenario.

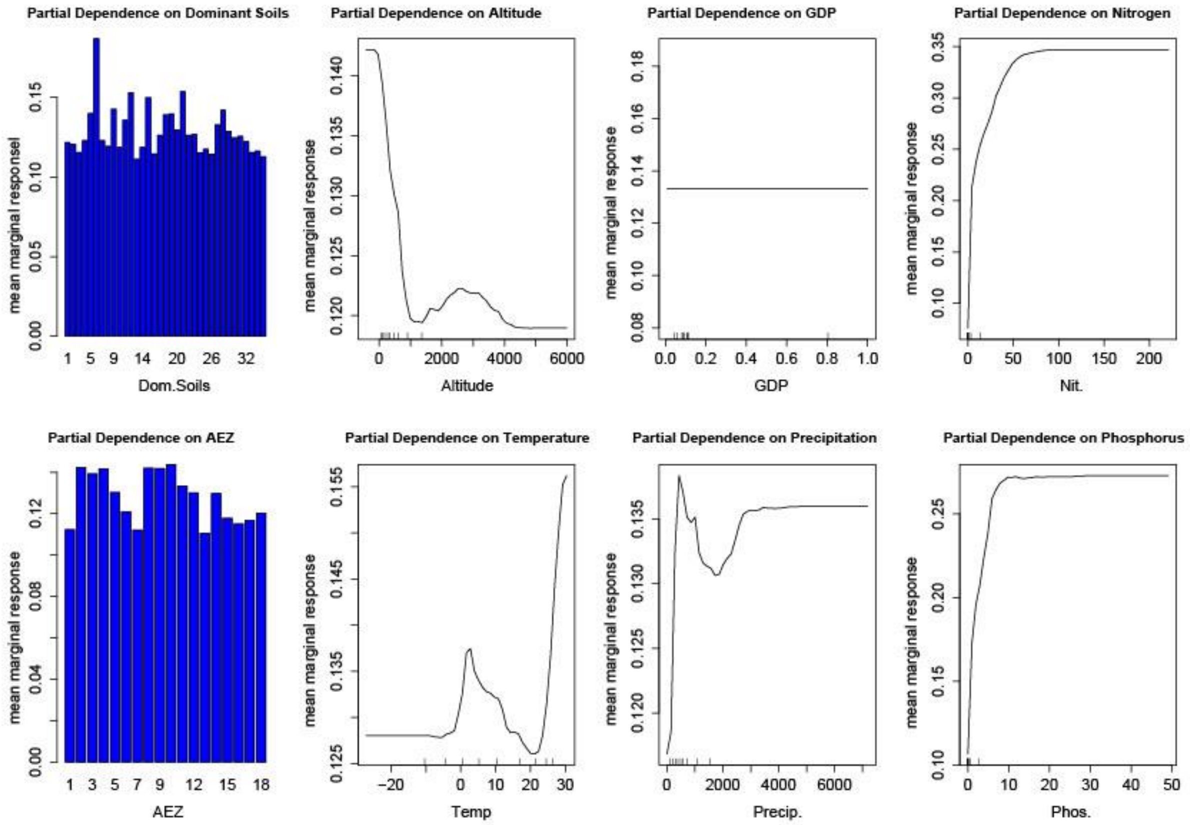

Figure S7. Partial plots for the trained Random Forest model.

References

Cai, Y., D. Newth, J. Finnigan, and D. Gunasekera. 2015. A hybrid energy-economy model for global integrated assessment of climate change, carbon mitigation and energy transformation. *Applied Energy* 148: 381–395. doi:10.1016/j.apenergy.2015.03.106.

Dix, M., P. Vohralik, and D. Bi. 2013. The ACCESS Coupled Model: Documentation of core CMIP5 simulations and initial results. *Australian Meteorological and Oceanographic Journal* 63: 83–99.

Fischer, G., and F. Nachtergaele. 2008. *Global agro-ecological zones assessment for agriculture (GAEZ 2008). IIASA, Laxenburg, Austria*.

Forster, P. M., T. Andrews, P. Good, J. M. Gregory, L. S. Jackson, and M. Zelinka. 2013. Evaluating adjusted forcing and model spread for historical and future scenarios in the CMIP5 generation of climate models. *Journal of Geophysical Research: Atmospheres* 118: 1139–1150. doi:10.1002/jgrd.50174.

Jones, P., and I. Harris. 2008. CRU Time Series (TS) high resolution gridded datasets. *University of East Anglia Climatic Research Unit (CRU) …*.

Liaw, A., and M. Wiener. 2002. Classification and regression by randomForest. *R news*.

Potter, P., and N. Ramankutty. 2010. Characterizing the spatial patterns of global fertilizer application and manure production. *Earth Interactions* 14: 1–22.

Ramankutty, N., and J. A. Foley. 1999. Estimating historical changes in global land cover: Croplands from 1700 to 1992. *Global Biogeochemical Cycles* 13: 997–1027.

Taylor, K. E., R. J. Stouffer, and G. A. Meehl. 2012. An Overview of CMIP5 and the Experiment Design. *Bulletin of the American Meteorological Society* 93: 485–498.

UN. 2012. *Probabilistic Population Projections - based on the 2012 Revision of the World Population Prospects*.

Vuuren, D. Van, J. Edmonds, and M. Kainuma. 2011. The representative concentration pathways: an overview. *Climatic Change*.
